# Golden Promise-rapid, a fast-cycling and transformable barley genotype

**DOI:** 10.1093/jxb/erag197

**Published:** 2026-04-22

**Authors:** Gabriele Buchmann, Einar Baldvin Haraldsson, Rebekka Schüller, Thea Rütjes, Agatha Alexandra Walla, Maria von Korff, Shanda Liu

**Affiliations:** Institute of Plant Genetics, Heinrich-Heine-Universität Düsseldorf, D-40225 Düsseldorf, Germany; Institute of Plant Genetics, Heinrich-Heine-Universität Düsseldorf, D-40225 Düsseldorf, Germany; Institute of Plant Genetics, Heinrich-Heine-Universität Düsseldorf, D-40225 Düsseldorf, Germany; Institute of Plant Genetics, Heinrich-Heine-Universität Düsseldorf, D-40225 Düsseldorf, Germany; Institute of Plant Genetics, Heinrich-Heine-Universität Düsseldorf, D-40225 Düsseldorf, Germany; Cluster of Excellence on Plant Sciences, ‘SMART Plants for Tomorrow’s Needs’, Heinrich-Heine-Universität Düsseldorf, D-40225 Düsseldorf, Germany; Institute of Plant Genetics, Heinrich-Heine-Universität Düsseldorf, D-40225 Düsseldorf, Germany; Cluster of Excellence on Plant Sciences, ‘SMART Plants for Tomorrow’s Needs’, Heinrich-Heine-Universität Düsseldorf, D-40225 Düsseldorf, Germany; Institute of Plant Genetics, Heinrich-Heine-Universität Düsseldorf, D-40225 Düsseldorf, Germany; Adelaide University, Australia

**Keywords:** *Agrobacterium*-mediated transformation, barley, fast-cycling, Golden Promise, Golden Promise-rapid, isogenic line, Ppd-H1, speed breeding, tissue culture

## Abstract

The spring barley cultivar Golden Promise (GP) is the major reference genotype for transformation due to its high transformability and the availability of a reference genome. However, GP is characterized by a long generation cycle and stress susceptibility under non-optimal growth conditions because it carries a mutation at the floral inducer *Photoperiod-H1* (*Ppd-H1*). Previously, we showed that a GP introgression line, Golden Promise-fast (GP-fast), generated by introducing the wild-type *Ppd-H1* allele from the winter barley cultivar Igri, exhibits early flowering and improved stress resilience. In this study, we generated a fast-cycling genotype, Golden Promise-rapid (GP-rapid), isogenic to GP with good regeneration capacity and transformability. We conducted two backcrosses of GP-fast to reduce the residual Igri genome. The resulting genotype contains only a single introgression of ∼0.6 Mbp at the *Ppd-H1* locus on chromosome 2H. Under speed-breeding conditions, its generation time was reduced to 63 d (25% shorter than the 84 d for GP). Parallel transformation of GP, GP-fast, and GP-rapid using CRISPR/Cas9-mediated genome editing of *Ppd-H1* revealed that GP-rapid remains amenable to *Agrobacterium*-mediated transformation. Overall, we report on the development of a fast-cycling GP isogenic line as a research tool for efficient generation of transgenic and gene-edited barley plants.

## Teaser

GP-rapid, a newly developed fast-cycling barley genotype, reduces generation time by 25% while remaining amenable to transformation, advancing functional genomic studies in barley.

## Introduction

Barley (*Hordeum vulgare*) is one of the major cereal crops and has emerged as a key genetic model for the *Triticeae* tribe. Its diploid genome, abundance of genetic resources, availability of a pan-genome, and transformation amenability make it particularly valuable for functional genomics (Saisho and Takeda, 2011; Harwood, 2019; Jayakodi *et al*., 2020). Recent advances in genome editing technologies have enabled the introduction of precise genetic modifications in barley (Han *et al*., 2021; Lawrenson *et al*., 2021, 2024; Tamilselvan-Nattar-Amutha *et al*., 2023), providing a powerful tool for dissecting causal relationships between genotype and phenotype.

Golden Promise (GP), a γ-ray-induced mutant of the cultivar Maythorpe, has served as the reference genotype for barley transformation for over two decades because it exhibits strong regeneration capacity in tissue culture and can achieve average transformation efficiencies of 25% with an *Agrobacterium*-mediated transformation protocol (Forster, 2001; Murray *et al*., 2004; Hensel *et al*., 2008; Harwood, 2012). Transformation attempts using other barley varieties have either failed or delivered low transformation efficiencies (Hensel *et al*., 2008; Harwood, 2012; Marthe *et al*., 2015). The use of GP for transformation and genome editing is further supported by the recent publication of a full reference genome (Jayakodi *et al*., 2020; Schreiber *et al*., 2020).

Despite these advantages, GP is also characterized by high environmental plasticity and a long generation cycle. GP carries a loss-of-function mutation in HvDep1, the Gγ subunit of a heterotrimeric G protein that positively regulates culm elongation and seed size in barley (Wendt *et al*., 2016). Wendt *et al*. demonstrated that the *HvDep1* loss-of-function mutation conferred either an increase or a decrease in harvestable yield depending on the environment and genetic background, thereby enhancing environmental plasticity. Previous studies reported that using stress-free explants is critical for achieving high transformation efficiency in GP (Harwood, 2014; Hayta *et al*., 2021). Its environmental plasticity poses challenges when donor plants are grown in non-optimal conditions. Additionally, as a spring barley, GP does not require vernalization to flower due to a deletion in the first intron of the vernalization gene *Vrn-H1* (5H) and a deletion of all three ZCCT copies underlying the *Vrn-H2* locus on 4H (Cockram *et al*., 2007). GP also carries a natural mutation at the major photoperiod response gene *Ppd-H1*, which causes a delay in reproductive development under long-day (LD) conditions (Turner *et al*., 2005). When grown under controlled conditions with 16 h light, GP requires ∼60–70 d to flower and 4 months to reach maturity (Campoli *et al*., 2012; Mulki *et al*., 2018; Pieper *et al*., 2021), limiting the number of generations to three per year. The long generation cycle of GP limits its suitability for the rapid generation of transgenic and gene-edited plants.

We previously showed that introgressing the wild-type *Ppd-H1* allele from the winter barley cultivar Igri into GP accelerated flowering time by 2 weeks under LD conditions, thereby considerably shortening the generation time (Gol *et al*., 2021). Furthermore, the resulting introgression line, termed GP-fast, displayed improved culm elongation and higher developmental stability and spike fertility in response to heat and drought, in contrast to GP (Gol *et al*., 2021; Lan *et al*., 2025). GP-fast has been successfully used for generating CRISPR/Cas9 [clustered regularly interspaced palindromic repeats (CRISPR)/CRISPR-associated protein 9] mutant lines (Amanda *et al*., 2022; Müller *et al*., 2023; Wanke *et al*., 2023; Helmsorig *et al*., 2024; Lan *et al*., 2025; Vardanega *et al*., 2025).

However, GP-fast retains at least three major Igri-derived genomic introgressions: one flanking the *Ppd-H1* locus on chromosome (Chr) 2H, and two others on Chr 6H and Chr 7H (Gol *et al*., 2021). This might become problematic for designing genome editing guides because nucleotide differences between the introgression line and the GP reference sequence can reduce guide accuracy and may also affect the performance of resulting transgenic lines. Furthermore, the introgressions on 2H and 6H partially overlap with minor quantitative trait loci (QTLs) affecting the transformation amenability in GP (Hisano and Sato, 2016; Hisano *et al*., 2017).

In this work, we greatly reduced the donor genome fractions in GP-fast by performing two additional rounds of backcrosses to GP. Genotyping results showed that the newly generated genotype, GP-rapid, contains only a 0.6 Mbp Igri introgression surrounding the *Ppd-H1* locus on Chr 2H with only 26 genes within this region. GP-rapid completes one generation in 63 d under speed-breeding conditions—a 25% reduction compared with GP. Importantly, GP-rapid retains the regeneration capacity and transformability of GP under our experimental conditions.

## Materials and methods

### Plant materials

Barley (*H. vulgare*) genotypes used in this study included GP, one selected GP-fast line (GP-fast_9, a sister line to the genotype described in Gol *et al*., 2021), and the newly developed line, GP-rapid. GP-fast was generated by crossing GP with the winter barley cultivar Igri, followed by three cycles of backcrossing and subsequent selfing (BC3F5) to obtain a homozygous line carrying the wild-type *Ppd-H1* allele. Chromosome-scale sequence assemblies of GP and Igri have been generated and compared within the barley pangenome project (Jayakodi *et al*., 2020).

GP, Igri, and the selected GP-fast line were first genotyped using the Barley 50k iSelect SNP Array (TraitGenetics GmbH, Germany) (Bayer *et al*., 2017). The BARLEYMAP pipeline was used to assign the single nucleotide polymorphism (SNP) array markers on the genetic map, POPSEQ_2017, and the physical map, the MorexV3 reference genome (Supplementary Fig. S1; Supplementary Table S1) (Cantalapiedra *et al*., 2015; Mascher *et al*., 2017, 2021). The donor introgression sizes for both genetic and physical positions were calculated based on the midpoint distance between a background (GP) SNP and the flanking distal SNP of the donor introgression (Igri) (Supplementary Table S2).

GP-rapid was generated by further backcrossing the selected GP-fast line (BC3F5) to GP, followed by four generations of selfing with marker-assisted selection (MAS) (BC4F5). One plant from the BC4F5 population was backcrossed one additional time to GP, followed by one generation of MAS-assisted selfing (BC5F2). In the resulting BC5F2 generation, one homozygous BC5F2 line was further validated using RNA-seq-based introgression genotyping (see below). This validated line was designated GP-rapid (Supplementary Fig. S2). MAS was conducted using cleaved amplified polymorphic sequence (CAPS) assays designed based on the Barley 50k iSelect SNP Array markers that differentiated the donor (Igri) and background (GP) genomes. CAPS markers were selected to cover the introgressed regions identified in GP-fast, with increased density on Chr 2H flanking the *Ppd-H1* locus (Supplementary Table S3).

### Genotyping using RNA-seq

Total RNA was extracted from leaf samples of the selected GP-rapid line, using the Qiagen RNeasy Plant Mini Kit (Qiagen, Hilden, Germany, Cat. No./ID: 74904), with the addition of 0.2% (v/v) β-mercaptoethanol. RNA quantity and quality were assessed using a NanoPhotometer NP80 (IMPLEN, Germany) and a 1% agarose gel. Purified total RNA samples were sequenced at Novogene UK (Cambridge, UK). Libraries were prepared by poly(A) tail capture, yielding 21 million Illumina PE150 reads (Supplementary Table S4). RNA-seq data for GP and GP-fast were previously reported (Lan *et al*., 2025).

The RNA-seq reads were quality-controlled with FastQC (v. 0.12.1) and their reports collated with MulitQC (v. 1.12) (Andrews, 2010; Ewels *et al*., 2016). All samples passed quality control, and the RNA-seq reads were aligned to the GP genome using STAR (v. 2.7.11a) aligner (Dobin *et al*., 2013; Schreiber *et al*., 2020). The following commands were applied: Genome indexing—‘STAR --runMode genomeGenerate --genomeFastaFiles $genome --sjdbGTFfile $gtf --sjdbOverhang 149; RNA-seq alignment—‘STAR --outSAMtype BAM SortedByCoordinate --outFilterMultimapNmax 1—outSAMmultNmax 1’.

The aligned reads (BAM) were curated with Samtools (v. 1.19.0) before calling SNPs with BCFtools (v. 1.19.0) (Danecek *et al*., 2021). The following commands were used: ‘samtools collate -O $BAM | samtools fixmate -m -- | samtools sort --o $sorted.BAM’, and SNPs were then called: ‘bcftools mpileup --redo-BAQ --min-BQ 30 --per- sample-mF -a AD,DP -d 1000 -f $genome -b $samples.list -Ou | bcftools call –multiallelic-caller –variants-only -Ob>out.sorted.bcf; bcftools view -i ‘QUAL>20' out.sorted.bcf>out.sorted.Q20.vcf’.

SNP calls in the introgression lines (GP-fast and GP-rapid) were compared with the allele called in the GP line used for backcrossing; SNPs matching the GP background were assigned 1 and 0 for the alternative allele (Supplementary Table S5). The sizes of donor introgressions were calculated as before, using the midpoint distance between GP and the alternative allele. The distal SNPs of introgressed regions were calculated based on a 5 Mbp rolling window that contained ≥10 SNPs that differed between the introgression lines and GP, and introgression windows closer than 15 Mbp were joined (Supplementary Table S6). Alternative allele SNP densities and introgressions were visualized and highlighted with karyoploteR (v. 1.30.0) in R (v. 4.4.3) (Gel and Serra, 2017; R Core Team, 2024).

### Growth conditions

Unless otherwise specified, plants were grown in 11 × 11 × 12 cm pots filled with the following standard substrate: Einheitserde ED73 (Einheitserde Werkverband e.V., Germany) was mixed with 7% sand and 4 g l^−1^ Osmocote Exact Hi End, 3–4M, fourth generation (ICL Group Ltd, UK). After sowing, seeds were stratified for 3 d at 4 °C and then transferred to climate-controlled growth chambers under the following conditions until maturity: 16 h light at 20 °C with photosynthetically active radiation (PAR) of 250 µmol m^−2^ s^−1^; 8 h dark at 16 °C.

### Speed-breeding conditions

Speed breeding was performed following a modified protocol based on Watson *et al*. (2018). Grains were first sown in 96-well trays with the standard substrate and stratified for 5 d at 4 °C. Seedlings were then grown under extended photoperiod conditions: 22 h light at 20 °C with PAR of 250 µmol m^−2^ s^−1^; 2 h dark at 16 °C. After 15 d of cultivation, seedlings were transplanted to 11 × 11 × 12 cm pots filled with standard substrate and grown to maturity.

One immature spike from each plant was harvested 3 weeks post-awn tipping (Zadoks stage 49, Zadoks *et al*., 1974) and immediately dried at 34 °C in an air-forced oven for 4 d. Germination assays were then conducted by placing 20 grains on wetted filter papers in a Petri dish. Six dishes were prepared for each genotype. After stratifying at 4 °C for 4 d, the Petri dishes were transferred to a dark incubator at 24 °C. Germination was assessed at 24 h and 48 h.

### Phenotypic analysis

Flowering time was recorded as the number of days after sowing (DAS) until awns emerged from the flag leaf sheath of the main culm, named awn tipping (Zadoks stage 49, Zadoks *et al*., 1974). Spike length, final floret number, and grain number were scored at maturity. Spike fertility was calculated as the ratio of grain number to the final floret number. Three spikes were measured for each plant.

### *Agrobacterium*-mediated barley transformation and genotyping

The binary vector construct employed to assess transformation efficiency was designed for mutagenizing the pseudo-receiver domain of Ppd-H1 (Turner *et al*., 2005), using CRISPR/Cas9. The construct was cloned based on a multiplex genome editing system (Kumar *et al*., 2018), with two single guide RNAs (sgRNAs) targeting the beginning of the third exon (spacer sequence: 5′-CCCCGTCGAGAACGGCCACC-3′) and fourth exon (spacer sequence: 5′-GATGTCGCACGATTCCA-3′), respectively. This construct has not been previously published, and an annotated sequence file (GenBank format) is available in Supplementary Dataset S1.

Barley immature embryo transformations followed a previously published protocol (Hensel *et al*., 2008), using *Agrobacterium* strain AGL1. Briefly, immature embryos (1.5–2 mm in diameter) were isolated and inoculated with *Agrobacterium* carrying the binary vector, then briefly washed and incubated at 21 °C in the dark in co-culture medium (CCM). After 3 d, embryos were transferred to callus induction medium (CIM) containing hygromycin (20 mg l^−1^) for selection and incubated at 24 °C in the dark. Embryos (calli) were moved to fresh CIM for an additional 2 weeks and subsequently to regeneration medium (RGM) with reduced hygromycin (10 mg l^−1^) under light conditions. Following two rounds of cultivation on RGM (2 weeks each), regenerated plantlets were transferred to rooting medium (RM) containing 20 mg l^−1^ hygromycin. Approximately 2 weeks later, plantlets were screened via PCR for successful T-DNA insertion (presence of *Cas9*). The following primers were used: 5′-GAGCGCATGAAGAGGATCGA-3′ and 5′-GGACACGAGCTTGGACTTGA-3′. Transgene-positive plantlets were transferred to RM without hygromycin for cultivation and photographing.

### Statistical analysis

Statistical tests were conducted using R (v. 4.4.3) (R Core Team, 2024). Student’s *t*-test was used for testing the significance between two groups (days to awn tipping), with a *P*-value cut-off at ≤0.01. For significance tests among three groups (days to awn tipping, spike fertility, spike length, and transformation efficiency), one-way ANOVA (function aov) and subsequent Tukey’s HSD (function TukeyHSD from library multcompView, v. 0.1.10) were used, with a *P*-value cut-off at ≤0.05 or ≤0.01. Replicate numbers are indicated in the corresponding figure legends. Plots were generated using ggplot2 (v. 3.5.2) (Wickham, 2016).

## Results

### GP-rapid retains one introgression of approximately 0.6 Mbp flanking the *Ppd-H1* allele

**Fig. 1.**
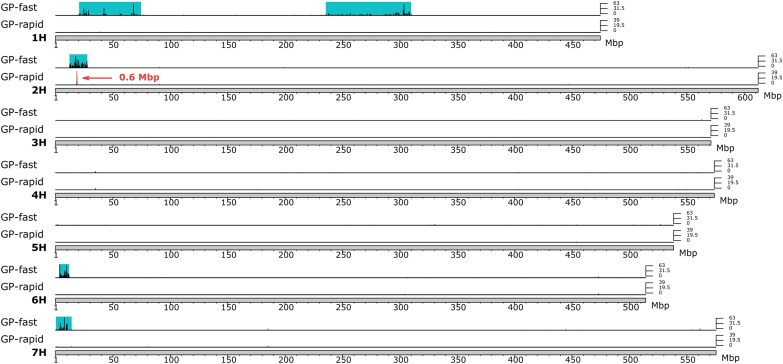
Genotyping by RNA-sequencing of Igri introgressions in GP-fast and GP-rapid. Density of SNPs differing between GP and GP-fast (top) and GP-rapid (bottom) on the seven chromosomes of the GP genome reference; SNP density is indicated in 10 kbp windows. Introgression regions are highlighted with rectangular boxes, GP-fast (cyan) and GP-rapid (red, location of the only introgression surrounding the *Ppd-H1* locus).

Genotyping of GP-fast with the Barley 50k iSelect SNP array revealed introgressions on chromosomes 1H (41.15–48.64 cM), 2H (flanking the *Ppd-H1* locus, 12.18–41.65 cM), 6H (5.84–24.58 cM), and 7H (1.53–21.52 cM) from the Igri genome (Supplementary Fig. S1; Supplementary Tables S1, S2). To reduce the identified introgressions, we next conducted two additional rounds of backcrossing to GP guided by MAS using CAPS markers (Supplementary Fig. S2; Supplementary Table S3). From the BC5F2 generation, a single plant was selected—hereafter referred to as GP-rapid—that only contained a single homozygous introgression surrounding the *Ppd-H1* locus on Chr 2H. RNA sequencing confirmed that the selected GP-rapid line contained only a single introgression on Chr 2H flanking the *Ppd-H1* locus, which was reduced from 16 Mbp to ∼0.6 Mbp (Chr 2H: 18 217 415–18 825 119) (Fig. 1; Supplementary Table S6). A total of 26 genes and two long terminal repeats (LTRs) were located within the introgressed interval. Their names, genomic positions, and putative gene functions are detailed in Table 1. Collectively, via MAS backcrossing, we generated a isogenic line GP-rapid, with an introgression surrounding *Ppd-H1* of 0.6 Mbp, including only 26 genes.

**Table 1.** List of genes within the retaining introgression in GP-rapid

Gene ID	Gene location (bp)	Putative gene function
**Horvu_GOLDEN_2H01G071200**	18216628–18218107	Pectate lyase
**Horvu_GOLDEN_2H01G071300**	18222613–18223779	Ta11-like non-LTR retrotransposon
**Horvu_GOLDEN_2H01G071400**	18225883–18228960	LPS-induced tumour necrosis factor alpha factor
**Horvu_GOLDEN_2H01G071500**	18239054–18241586	Actin cross-linking protein
**Horvu_GOLDEN_2H01G071600**	18285599–18287394	Transcription factor
**Horvu_GOLDEN_2H01G071700**	18293972–18296679	Protein kinase-like protein
**Horvu_GOLDEN_2H01G071800**	18346036–18346605	RING/U-box superfamily protein
**Horvu_GOLDEN_2H01G071900**	18351304–18354525	Pseudo-response regulator (Ppd-H1)
**Horvu_GOLDEN_2H01G072000**	18366492–18367622	Strictosidine synthase
**Horvu_GOLDEN_2H01G072100**	18394728–18399897	Sporulation protein RMD1
**Horvu_GOLDEN_2H01G072200**	18401162–18403746	Ascorbate peroxidase
**Horvu_GOLDEN_2H01G072300**	18404602–18407365	Arogenate dehydratase
**Horvu_GOLDEN_2H01G072400**	18444402–18446931	Myb/SANT-like DNA-binding domain protein
**Horvu_GOLDEN_2H01G072500**	18452826–18453248	Tapetum determinant 1
**Horvu_GOLDEN_2H01G072600**	18477588–18480133	UvrABC system protein C
**Horvu_GOLDEN_2H01G072700**	18482260–18484041	Serine/threonine-protein kinase ATM
**Horvu_GOLDEN_2H01G072800**	18500675–18502613	Glycosyltransferase
**Horvu_GOLDEN_2H01G072900**	18504006–18507914	Glycosyltransferase
**Horvu_GOLDEN_2H01G073000**	18567774–18569554	Elongation factor 1-alpha
**Horvu_GOLDEN_2H01G073100**	18633447–18635137	Retrotransposon protein, putative, unclassified
**Horvu_GOLDEN_2H01G073200**	18641793–18642503	Leucine-rich repeat receptor-like protein kinase family protein
**Horvu_GOLDEN_2H01G073300**	18675353–18677359	Elongation factor 1-alpha
**Horvu_GOLDEN_2H01G073400**	18679940–18684042	Yellow stripe-like transporter 12
**Horvu_GOLDEN_2H01G073500**	18692529–18693696	Retrovirus-related Pol polyprotein from transposon TNT 1-94
**Horvu_GOLDEN_2H01G073600**	18738300–18741443	MLO-like protein
**Horvu_GOLDEN_2H01G073700**	18763915–18766983	MLO-like protein
**Horvu_GOLDEN_2H01G073800**	18816650–18817055	GRF zinc finger family protein
**Horvu_GOLDEN_2H01G073900**	18817600–18820898	Phosphoinositide phospholipase C

### GP-rapid displays early flowering and improved spike fertility

**Fig. 2.**
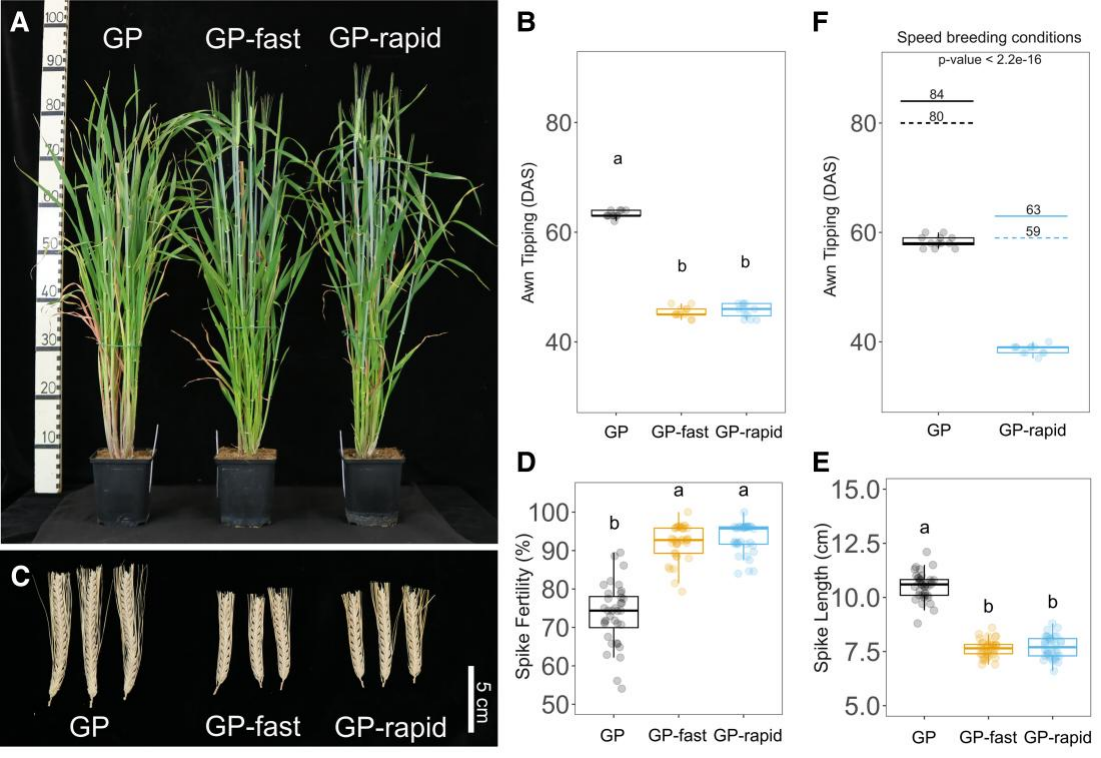
Phenotypic comparisons of GP, GP-fast, and GP-rapid. Phenotypic comparisons of GP, GP-fast, and GP-rapid under long-day (16 h light/8 h dark) conditions (A–E) and speed-breeding (22 h light/2 h dark) conditions (F). (A) Representative plants 61 days after sowing (DAS). Fully emerged spikes can be observed for GP-fast and GP-rapid, whereas awns are not yet visible for GP. (B) Number of days between sowing and awn tipping, *n*=12. (C) Spike morphology at maturity, scale bar=5 cm. (D) Spike fertility (no. of grains/no. of florets in percentage), and (E) spike length, *n*=36. (F) Awn tipping and growing cycle comparison between GP and GP-rapid under speed-breeding conditions. Dashed lines indicate when immature spikes were harvested (3 weeks post-awn tipping), and the solid lines represent when viable grains were obtained (dried at 34 °C for 4 d), *n*=12. For (B), (D), and (E), the data were analysed by a one-way ANOVA and Tukey’s honestly significant difference (HSD). Error bars represent the SD, *P*-values <0.01. For (F), Student’s *t*-test was employed.

We compared the developmental traits of GP-rapid with those of GP and GP-fast. All plants were grown under LD conditions with 16 h light/8 h dark. GP-rapid displayed an early flowering phenotype compared with GP (Fig. 2A). Awn tipping (Zadoks stage 49, Zadoks *et al*., 1974) in GP-rapid occurred on average 45.7 DAS, 18 d earlier than GP, but was not significantly different from GP-fast, which flowered on average 45.4 DAS (Fig. 2B). Additionally, we also observed that GP-rapid and GP-fast had comparable and shorter spikes (7.6±0.4 cm and 7.7±0.5 cm, respectively) than GP (10.5±0.6 cm) (Fig. 2C, E). Despite a reduction in spike length, GP-rapid displayed a higher spike fertility (93.2±3.9%) compared with GP (73.4±8.0%), similar to GP-fast (92.1±4.6%) (Fig. 2C, D). These observations are consistent with previous reports, indicating that the wild-type *Ppd-H1* allele is associated with higher spike fertility under different conditions (Digel *et al*., 2015; Ejaz and von Korff, 2017; Gol *et al*., 2021; Lan *et al*., 2025).

Overall, these findings show that GP-rapid is early flowering and displays higher spike fertility than GP, providing further evidence that the *Ppd-H1* allele is primarily responsible for the trait differences described for GP-fast.

### GP-rapid exhibits a 25% shorter growth cycle under speed-breeding conditions

The advent of speed-breeding strategies has further revolutionized crop development timelines by significantly reducing generation times in various crops, including barley (Watson *et al*., 2018). These methods combine extended photoperiods (e.g. 22 h light/2 h dark) and the use of pre-harvest immature spikes, enabling up to six generations per year, compared with the conventional three generations under standard growth conditions (Watson *et al*., 2018). Two genes, *Ppd-H1* and *ELF3,* have been identified as key contributors to accelerated development under speed-breeding conditions (Rossi *et al*., 2024). Given that GP-rapid carries the wild-type *Ppd-H1* allele, its flowering time and generation time could probably be further reduced through speed-breeding protocols.

To test this, we further compared flowering time and seed-to-seed cycle time between GP-rapid and GP under speed-breeding conditions. Our results showed that under extended photoperiods (22 h light/2 h dark), awn tipping in GP-rapid took place 38.6 DAS (Fig. 2F), ∼7 d sooner than when grown under 16 h light/8 h dark (45.7 d, Fig. 2B). GP, however, reached the same developmental stage ∼20 d later than GP-rapid under speed-breeding condition (58.3 d, Fig. 2F). Both genotypes progressed to early dough stage (Zadoks stage 83, Zadoks *et al*., 1974) 3 weeks post-awn tipping, at which point immature spikes were harvested (Fig. 2F, dashed lines), dried at 34 °C for 4 d, and subjected to germination tests (Fig. 2F, solid lines). Germination rates exceeded 80% in both genotypes within 48 h (Table 2), confirming the viability of the harvested seeds.

**Table 2.** Germination test of grains harvested from the speed-breeding trial

Genotype	Germination after 24 h (%)	Germination after 48 h (%)
**GP**	65.0±13.4 (*n*=6)	85.8±9.7 (*n*=6)
**GP-rapid**	55.8±15.6 (*n*=6)	82.5±12.1 (*n*=6)

In summary, the implementation of speed breeding enabled a complete seed-to-seed cycle in only 63 d for GP-rapid, representing a 25% reduction compared with 84 d for GP (Fig. 2F). This accelerated cycling enables up to six generations per year and positions GP-rapid as a highly efficient genotype for fast-forwarding genetic studies.

### GP-rapid is amenable to transformation

We next investigated whether GP-rapid maintains amenability to *Agrobacterium*-mediated immature embryo transformation as does GP. Previous studies identified transformation amenability (TFA) loci contributing to the high transformation amenability of GP (Hisano and Sato, 2016; Hisano *et al*., 2017; Orman-Ligeza *et al*., 2020). Since GP-rapid still retains a segment from the Igri genome on Chr 2H (Fig. 1), which is in proximity to one of the TFA loci (TFA5, Hisano *et al*., 2016), it was critical to evaluate the transformability of GP-rapid. To do so, we chose a CRISPR construct targeting the pseudo-receiver domain of Ppd-H1 and performed *Agrobacterium*-mediated transformation using immature embryos from GP and GP-rapid, as well as GP-fast. Four independent transformations were conducted. For each genotype, the total number of embryos was between 597 and 851 (Table 3).

**Table 3.** Transformation efficiencies using the Ppd-H1 CRISPR construct

Experiment	Genotype	Replicate	No. of embryos	Cas9-positive plants	Transformation efficiency (%)
**1**	GP-fast	No. 1	76	10	13.16
	GP-fast	No. 2	97	10	10.31
	GP-rapid	No. 1	95	4	4.22
	GP-rapid	No. 2	101	12	11.89
**2**	GP	No. 1	37	1	2.71
	GP	No. 2	42	0	0
	GP-fast	No. 1	66	1	1.52
	GP-fast	No. 2	106	3	2.84
	GP-rapid	No. 1	62	3	4.84
	GP-rapid	No. 2	66	5	7.58
**3**	GP	No. 1	98	4	4.09
	GP	No. 2	134	2	1.5
	GP-fast	No. 1	52	1	1.93
	GP-fast	No. 2	72	0	0
	GP-rapid	No. 1	94	1	1.07
	GP-rapid	No. 2	116	4	3.45
**4**	GP	No. 1	135	6	4.45
	GP	No. 2	151	2	1.33
	GP-fast	No. 1	69	2	2.9
	GP-fast	No. 2	76	2	2.64
	GP-rapid	No. 1	142	1	0.71
	GP-rapid	No. 2	175	5	2.86
**Total**	GP		**597**	15	2.52
	GP-fast		**614**	29	4.73
	GP-rapid		**851**	35	4.12

**Fig. 3.**
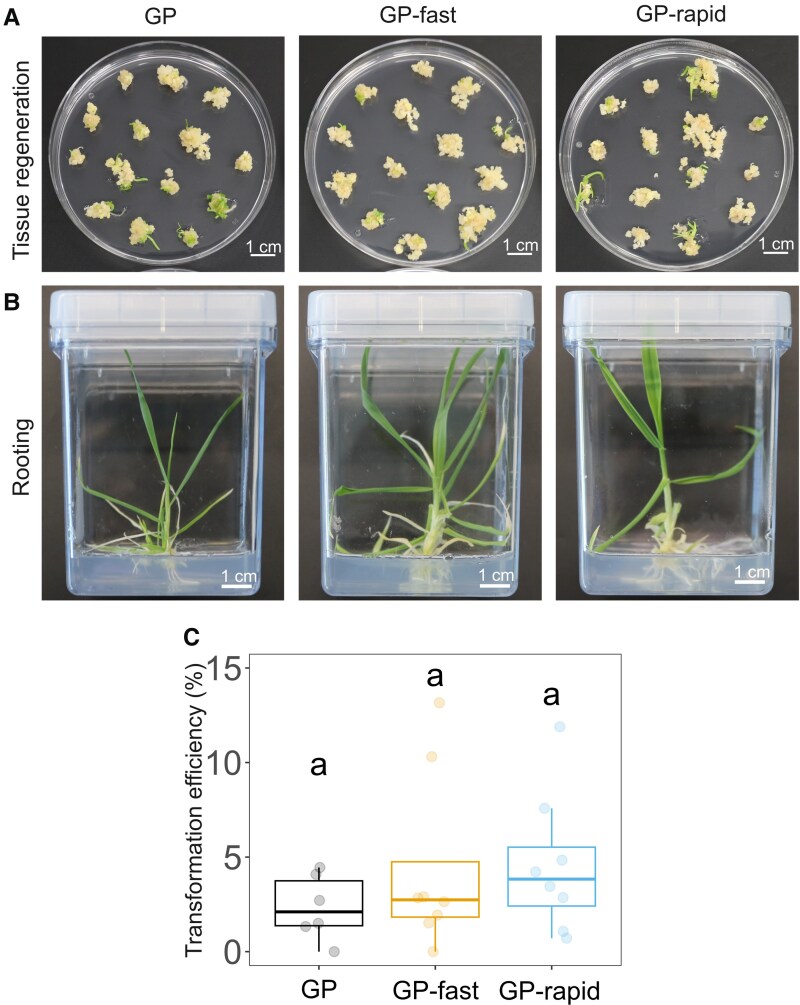
Regeneration and transformation amenability in GP, GP-fast, and GP-rapid. (A) Calli regeneration 14 d after transferring to regeneration medium (RGM) containing hygromycin; scale bars=1 cm. (B) Representative transgene-positive plants 8 d after transferring to rooting medium (RM) without hygromycin; scale bars=1 cm. (C) Transformation efficiency comparison across the three genotypes. Data points represent individual transformations, *n*=6–8. In each transformation, the number of explants varies from 37 to 175. A detailed summary of transformation results can be found in Table 3. The data were analysed by a one-way ANOVA and Tukey’s honestly significant difference (HSD). No significant difference was detected (*P*-values >0.05). Error bars represent the SD.

Efficient regeneration from explants is a critical requirement for successful transformation. In our trials, all three genotypes exhibited rapid plantlet regeneration from calli within 2 weeks on RGM containing hygromycin (selective agent) (Fig. 3A). Furthermore, transgenic plantlets developed healthy shoots and roots on the RM (Fig. 3B). Genotyping of T_0_ plants revealed comparable transformation efficiencies among the three genotypes (Fig. 3C; Table 3). In conclusion, GP-rapid remains amenable to immature embryo transformation.

## Discussion

Recent advancements in CRISPR/Cas-mediated genome editing technologies have facilitated precise and efficient targeted mutagenesis in numerous crop species, including barley (Gasparis *et al*., 2018; Lawrenson *et al*., 2021, 2024). GP, known for its high transformation amenability and well-characterized genome, has become the reference genotype for genetic transformation (Murray *et al*., 2004; Hensel *et al*., 2008; Harwood, 2012; Schreiber *et al*., 2020). In this study, we generated a fast-cycling GP isogenic homozygous line, named GP-rapid (Fig. 1). This genotype showed 25% shorter seed-to-seed cycle time compared with GP under speed-breeding conditions, enabling up to six generations per year (Fig. 2F). Importantly, the Igri introgression was significantly reduced relative to GP-fast, simplifying downstream applications such as guide RNA design for genome editing.

Beyond shortening the life cycle, the wild-type *Ppd-H1* allele in barley enhances resilience and stabilizes development and yield under abiotic stress conditions (Wiegmann *et al*., 2019; Gol *et al*., 2021; Lan *et al*., 2025). Furthermore, lines carrying the wild-type *Ppd-H1* allele maintained transcriptomic stability, in contrast to spring barley genotypes with the natural *ppd-H1* mutation, which activated a strong transcriptional stress response under high-temperature conditions (Lan *et al*., 2025). Prior reports have suggested that transformation success is influenced by the physiological condition of donor plants, with stress-free plants producing higher-quality explants (Harwood, 2014; Hayta *et al*., 2021). Therefore, GP-rapid may be particularly advantageous for transformation if donor or transgenic plants are grown in environments with suboptimal or variable growth conditions.

In conclusion, GP-rapid is a versatile and efficient genetic system for barley research. It combines a fast generation cycle, good transformability, and improved stress tolerance. Its adoption may substantially accelerate functional genomic studies and genome editing applications in barley.

## Supplementary Material

erag197_Supplementary_Data

## Data Availability

The FAIR data supporting the findings of this work are available at DataPLANT (Weil *et al*., 2023) under the DOI: https://doi.org/10.60534/tpmdy-zrc03.

## Supplementary data

The following supplementary data are available at *JXB* online.

Fig. S1. Barley 50k iSelect SNP array genotyping of the selected GP-fast line used for further backcrossing with GP.

Fig. S2. Graphic overview of GP-rapid generation.

Table S1. Igri introgressions in the selected single GP-fast line using the Barley 50k iSelect SNP array.

Table S2. Igri introgression sizes in the selected single GP-fast line based on the Barley 50k iSelect SNP array.

Table S3. List of CAPS markers used for marker-assisted genotype selection.

Table S4. RNA-seq libraries used for genotyping of introgressions.

Table S5. Igri introgression analysis using RNA-seq-based genotyping in the selected single GP-fast line and GP-rapid based on RNA-seq-based genotyping.

Table S6. Igri introgression sizes in the selected single GP-fast line and GP-rapid based on RNA-seq-based genotyping.

Dataset S1. An annotated sequence file of the plasmid used for testing transformation efficiency.

## References

[erag197-B1] Amanda D, Frey FP, Neumann U, et al 2022. Auxin boosts energy generation pathways to fuel pollen maturation in barley. Current Biology 32, 1798–1811. 35316655 10.1016/j.cub.2022.02.073

[erag197-B2] Andrews S . 2010. FastQC: a quality control tool for high throughput sequence data. Cambridge, UK: Babraham Bioinformatics.

[erag197-B3] Bayer MM, Rapazote-Flores P, Ganal M, et al 2017. Development and evaluation of a barley 50k iSelect SNP array. Frontiers in Plant Science 8, 1792. 29089957 10.3389/fpls.2017.01792 PMC5651081

[erag197-B4] Campoli C, Drosse B, Searle I, Coupland G, von Korff M. 2012. Functional characterisation of *HvCO1*, the barley (*Hordeum vulgare*) flowering time ortholog of *CONSTANS*. The Plant Journal 69, 868–880. 22040323 10.1111/j.1365-313X.2011.04839.x

[erag197-B5] Cantalapiedra CP, Boudiar R, Casas AM, Igartua E, Contreras-Moreira B. 2015. BARLEYMAP: physical and genetic mapping of nucleotide sequences and annotation of surrounding loci in barley. Molecular Breeding 35, 13.

[erag197-B6] Cockram J, Donini P, Chiapparino E, Laurie DA, Stamati K, Taylor SA, O’Sullivan DM. 2007. Haplotype analysis of vernalization loci in European barley germplasm reveals novel *VRN-H1* alleles and a predominant winter *VRN-H1/VRN-H2* multi-locus haplotype. Theoretical and Applied Genetics 115, 993–1001. 17713756 10.1007/s00122-007-0626-x

[erag197-B7] Danecek P, Pollard MO, Liddle J, et al 2021. Twelve years of SAMtools and BCFtools. GigaScience 10, . 10.1093/gigascience/giab008 PMC7931819 33590861

[erag197-B8] Digel B, Pankin A, von Korff M. 2015. Global transcriptome profiling of developing leaf and shoot apices reveals distinct genetic and environmental control of floral transition and inflorescence development in barley. The Plant Cell 27, 2318–2334. 26307377 10.1105/tpc.15.00203 PMC4815099

[erag197-B9] Dobin A, Davis CA, Schlesinger F, Drenkow J, Zaleski C, Jha S, Batut P, Chaisson M, Gingeras TR. 2013. STAR: ultrafast universal RNA-seq aligner. Bioinformatics 29, 15–21. 23104886 10.1093/bioinformatics/bts635 PMC3530905

[erag197-B10] Ejaz M, Von Korff M. 2017. The genetic control of reproductive development under high ambient temperature. Plant Physiology 173, 294–306. 28049855 10.1104/pp.16.01275 PMC5210726

[erag197-B11] Ewels P, Magnusson M, Lundin S, Käller M. 2016. MultiQC: summarize analysis results for multiple tools and samples in a single report. Bioinformatics 32, 3047–3048. 27312411 10.1093/bioinformatics/btw354 PMC5039924

[erag197-B12] Forster BP . 2001. Mutation genetics of salt tolerance in barley: an assessment of Golden Promise and other semi-dwarf mutants. Euphytica 120, 317–328.

[erag197-B13] Gasparis S, Kała M, Przyborowski M, Łyżnik LA, Orczyk W, Nadolska-Orczyk A. 2018. A simple and efficient CRISPR/Cas9 platform for induction of single and multiple, heritable mutations in barley (*Hordeum vulgare* L.). Plant Methods 14, 111. 30568723 10.1186/s13007-018-0382-8 PMC6297969

[erag197-B14] Gel B, Serra E. 2017. Karyoploter: an R/Bioconductor package to plot customizable genomes displaying arbitrary data. Bioinformatics 33, 3088–3090. 28575171 10.1093/bioinformatics/btx346 PMC5870550

[erag197-B15] Gol L, Haraldsson EB, von Korff M. 2021. *Ppd-H1* integrates drought stress signals to control spike development and flowering time in barley. Journal of Experimental Botany 72, 122–136. 32459309 10.1093/jxb/eraa261 PMC7816852

[erag197-B16] Han Y, Broughton S, Liu L, Zhang X-Q, Zeng J, He X, Li C. 2021. Highly efficient and genotype-independent barley gene editing based on anther culture. Plant Communications 2, 100082. 33898972 10.1016/j.xplc.2020.100082 PMC8060703

[erag197-B17] Harwood WA . 2012. Advances and remaining challenges in the transformation of barley and wheat. Journal of Experimental Botany 63, 1791–1798. 22140237 10.1093/jxb/err380

[erag197-B18] Harwood WA . 2014. A protocol for high-throughput *Agrobacterium*-mediated barley transformation. Methods in Molecular Biology 1099, 251–260. 24243209 10.1007/978-1-62703-715-0_20

[erag197-B19] Harwood WA . ed. 2019. Barley: methods and protocols. New York, NY: Springer New York.

[erag197-B20] Hayta S, Smedley MA, Clarke M, Forner M, Harwood WA. 2021. An efficient *Agrobacterium*-mediated transformation protocol for hexaploid and tetraploid wheat. Current Protocols 1, e58. 33656289 10.1002/cpz1.58

[erag197-B21] Helmsorig G, Walla A, Rütjes T, Buchmann G, Schüller R, Hensel G, von Korff M. 2024. *Early maturity 7* promotes early flowering by controlling the light input into the circadian clock in barley. Plant Physiology 194, 849–866. 37951242 10.1093/plphys/kiad551 PMC10828213

[erag197-B22] Hensel G, Valkov V, Middlefell-Williams J, Kumlehn J. 2008. Efficient generation of transgenic barley: the way forward to modulate plant–microbe interactions. Journal of Plant Physiology 165, 71–82. 17905476 10.1016/j.jplph.2007.06.015

[erag197-B23] Hisano H, Sato K. 2016. Genomic regions responsible for amenability to *Agrobacterium*-mediated transformation in barley. Scientific Reports 6, 37505. 27874056 10.1038/srep37505 PMC5118740

[erag197-B24] Hisano H, Meints B, Moscou MJ, Cistue L, Echávarri B, Sato K, Hayes PM. 2017. Selection of transformation-efficient barley genotypes based on TFA (transformation amenability) haplotype and higher resolution mapping of the TFA loci. Plant Cell Reports 36, 611–620. 28204911 10.1007/s00299-017-2107-2

[erag197-B25] Jayakodi M, Padmarasu S, Haberer G, et al 2020. The barley pan-genome reveals the hidden legacy of mutation breeding. Nature 588, 284–289. 33239781 10.1038/s41586-020-2947-8 PMC7759462

[erag197-B26] Kumar N, Galli M, Ordon J, Stuttmann J, Kogel K, Imani J. 2018. Further analysis of barley MORC 1 using a highly efficient RNA-guided Cas9 gene-editing system. Plant Biotechnology Journal 16, 1892–1903. 29577542 10.1111/pbi.12924 PMC6181210

[erag197-B27] Lan T, Walla A, Çolpan Karışan KE, et al 2025. *PHOTOPERIOD 1* enhances stress resistance and energy metabolism to promote spike fertility in barley under high ambient temperatures. Plant Physiology 197, kiaf118. 40139938 10.1093/plphys/kiaf118 PMC12002028

[erag197-B28] Lawrenson T, Hinchliffe A, Clarke M, Morgan Y, Harwood W. 2021. In-planta gene targeting in barley using Cas9 with and without geminiviral replicons. Frontiers in Genome Editing 3, 663380. 34713258 10.3389/fgeed.2021.663380 PMC8525372

[erag197-B29] Lawrenson T, Clarke M, Kirby R, Forner M, Steuernagel B, Brown JKM, Harwood W. 2024. An optimised CRISPR Cas9 and Cas12a mutagenesis toolkit for barley and wheat. Plant Methods 20, 123. 39138524 10.1186/s13007-024-01234-y PMC11321142

[erag197-B30] Marthe C, Kumlehn J, Hensel G. 2015. Barley (*Hordeum vulgare* L.) transformation using immature embryos. Methods in Molecular Biology 1223, 71–83. 25300832 10.1007/978-1-4939-1695-5_6

[erag197-B31] Mascher M, Gundlach H, Himmelbach A, et al 2017. A chromosome conformation capture ordered sequence of the barley genome. Nature 544, 427–433. 28447635 10.1038/nature22043

[erag197-B32] Mascher M, Wicker T, Jenkins J, et al 2021. Long-read sequence assembly: a technical evaluation in barley. The Plant Cell 33, 1888–1906. 33710295 10.1093/plcell/koab077 PMC8290290

[erag197-B33] Mulki MA, Bi X, von Korff M. 2018. FLOWERING LOCUS T3 controls spikelet initiation but not floral development. Plant Physiology 178, 1170–1186. 30213796 10.1104/pp.18.00236 PMC6236595

[erag197-B34] Müller Y, Patwari P, Stöcker T, et al 2023. Isolation and characterization of the gene *HvFAR1* encoding acyl-CoA reductase from the *cer-za.227* mutant of barley (*Hordeum vulgare*) and analysis of the cuticular barrier functions. New Phytologist 239, 1903–1918. 37349864 10.1111/nph.19063

[erag197-B35] Murray F, Brettell R, Matthews P, Bishop D, Jacobsen J. 2004. Comparison of *Agrobacterium*-mediated transformation of four barley cultivars using the GFP and GUS reporter genes. Plant Cell Reports 22, 397–402. 14530864 10.1007/s00299-003-0704-8

[erag197-B36] Orman-Ligeza B, Harwood W, Hedley PE, Hinchcliffe A, Macaulay M, Uauy C, Trafford K. 2020. TRA1: a locus responsible for controlling *Agrobacterium*-mediated transformability in barley. Frontiers in Plant Science 11, 355. 32373138 10.3389/fpls.2020.00355 PMC7176908

[erag197-B37] Pieper R, Tomé F, Pankin A, von Korff M. 2021. *FLOWERING LOCUS T4* delays flowering and decreases floret fertility in barley. Journal of Experimental Botany 72, 107–121. 33048122 10.1093/jxb/eraa466 PMC7816854

[erag197-B38] R Core Team . 2024. R: a language and environment for statistical computing. Vienna, Austria: R Foundation for Statistical Computing. https://www.R-project.org/

[erag197-B39] Rossi N, Powell W, Mackay IJ, Hickey L, Maurer A, Pillen K, Halliday K, Sharma R. 2024. Investigating the genetic control of plant development in spring barley under speed breeding conditions. Theoretical and Applied Genetics 137, 115. 38691245 10.1007/s00122-024-04618-9 PMC11063105

[erag197-B40] Saisho D, Takeda K. 2011. Barley: emergence as a new research material of crop science. Plant & Cell Physiology 52, 724–727. 21565909 10.1093/pcp/pcr049

[erag197-B41] Schreiber M, Mascher M, Wright J, Padmarasu S, Himmelbach A, Heavens D, Milne L, Clavijo BJ, Stein N, Waugh R. 2020. A genome assembly of the barley ‘transformation reference’ cultivar Golden Promise. G3 Genes|Genomes|Genetics 10, 1823–1827. 32241919 10.1534/g3.119.401010 PMC7263683

[erag197-B42] Tamilselvan-Nattar-Amutha S, Hiekel S, Hartmann F, Lorenz J, Dabhi RV, Dreissig S, Hensel G, Kumlehn J, Heckmann S. 2023. Barley stripe mosaic virus-mediated somatic and heritable gene editing in barley (*Hordeum vulgare* L.). Frontiers in Plant Science 14, 1201446. 37404527 10.3389/fpls.2023.1201446 PMC10315673

[erag197-B43] Turner A, Beales J, Faure S, Dunford RP, Laurie DA. 2005. The pseudo-response regulator *Ppd-H1* provides adaptation to photoperiod in barley. Science 310, 1031–1034. 16284181 10.1126/science.1117619

[erag197-B44] Vardanega I, Maika JE, Demesa-Arevalo E, et al 2025. CLAVATA signalling shapes barley inflorescence by controlling activity and determinacy of shoot meristem and rachilla. Nature Communications 16, 3937. 10.1038/s41467-025-59330-z PMC12033307 40287461

[erag197-B45] Wanke A, Van Boerdonk S, Mahdi LK, et al 2023. A GH81-type β-glucan-binding protein enhances colonization by mutualistic fungi in barley. Current Biology 33, 5071–5084.e7. 37977140 10.1016/j.cub.2023.10.048

[erag197-B46] Watson A, Ghosh S, Williams MJ, et al 2018. Speed breeding is a powerful tool to accelerate crop research and breeding. Nature Plants 4, 23–29. 29292376 10.1038/s41477-017-0083-8

[erag197-B47] Weil HL, Schneider K, Tschöpe M, et al 2023. PLANTdataHUB: a collaborative platform for continuous FAIR data sharing in plant research. The Plant Journal 116, 974–988. 37818860 10.1111/tpj.16474

[erag197-B48] Wendt T, Holme I, Dockter C, Preuß A, Thomas W, Druka A, Waugh R, Hansson M, Braumann I. 2016. Hvdep1 is a positive regulator of culm elongation and grain size in barley and impacts yield in an environment-dependent manner. PLoS One 11, e0168924. 28005988 10.1371/journal.pone.0168924 PMC5179111

[erag197-B49] Wickham H . 2016. ggplot2: elegant graphics for data analysis. New York: Springer-Verlag. https://ggplot2.tidyverse.org.

[erag197-B50] Wiegmann M, Maurer A, Pham A, et al 2019. Barley yield formation under abiotic stress depends on the interplay between flowering time genes and environmental cues. Scientific Reports 9, 6397. 31024028 10.1038/s41598-019-42673-1 PMC6484077

[erag197-B51] Zadoks JC, Chang TT, Konzak CF. 1974. A decimal code for the growth stages of cereals. Weed Research 14, 415–421.

