# Supplementary material for: Golden Promise-rapid, a fast-cycling and transformable barley genotype
Source: J Exp Bot. 2026 Apr 22;77(14):4328–36. doi: 10.1093/jxb/erag197 (PMC13415965; doi:10.1093/jxb/erag197)
Supplement: erag197_Supplementary_Data [file erag197_supplementary_data.zip › JEXBOT317022-file002.pdf]

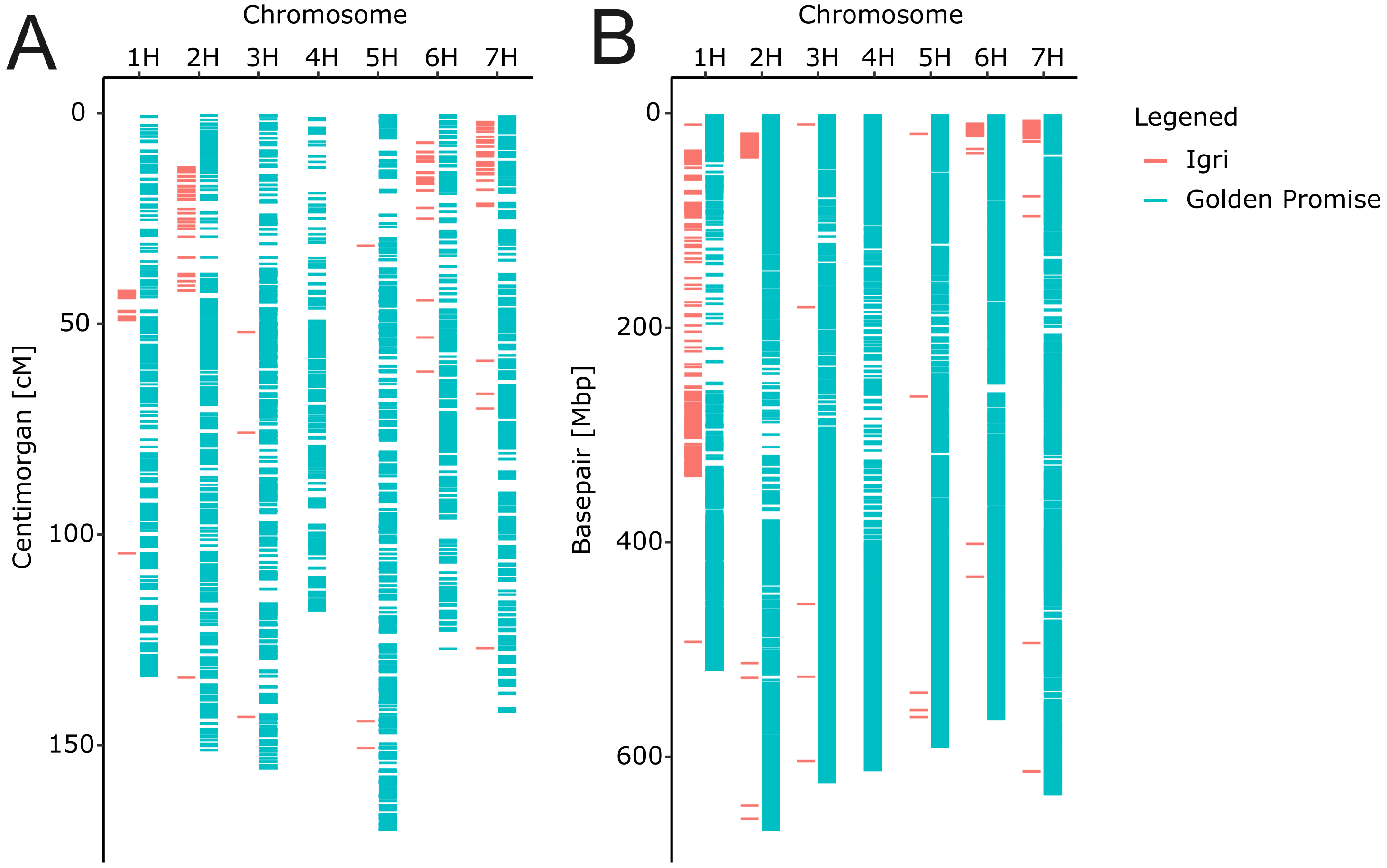

Fig. S1. Barley 50k iSelect SNP array genotyping of the selected GP-fast line (GP-fast_9) used for further backcrossing with GP. Markers are indicated for Igri (red, foreign material) and GP background in (blue). (A) Genetic map with marker location from POPSEQ_2017 and (B) physical map with marker physical location obtained from Morex V3 reference genome (Cantalapiedra et al., 2015).

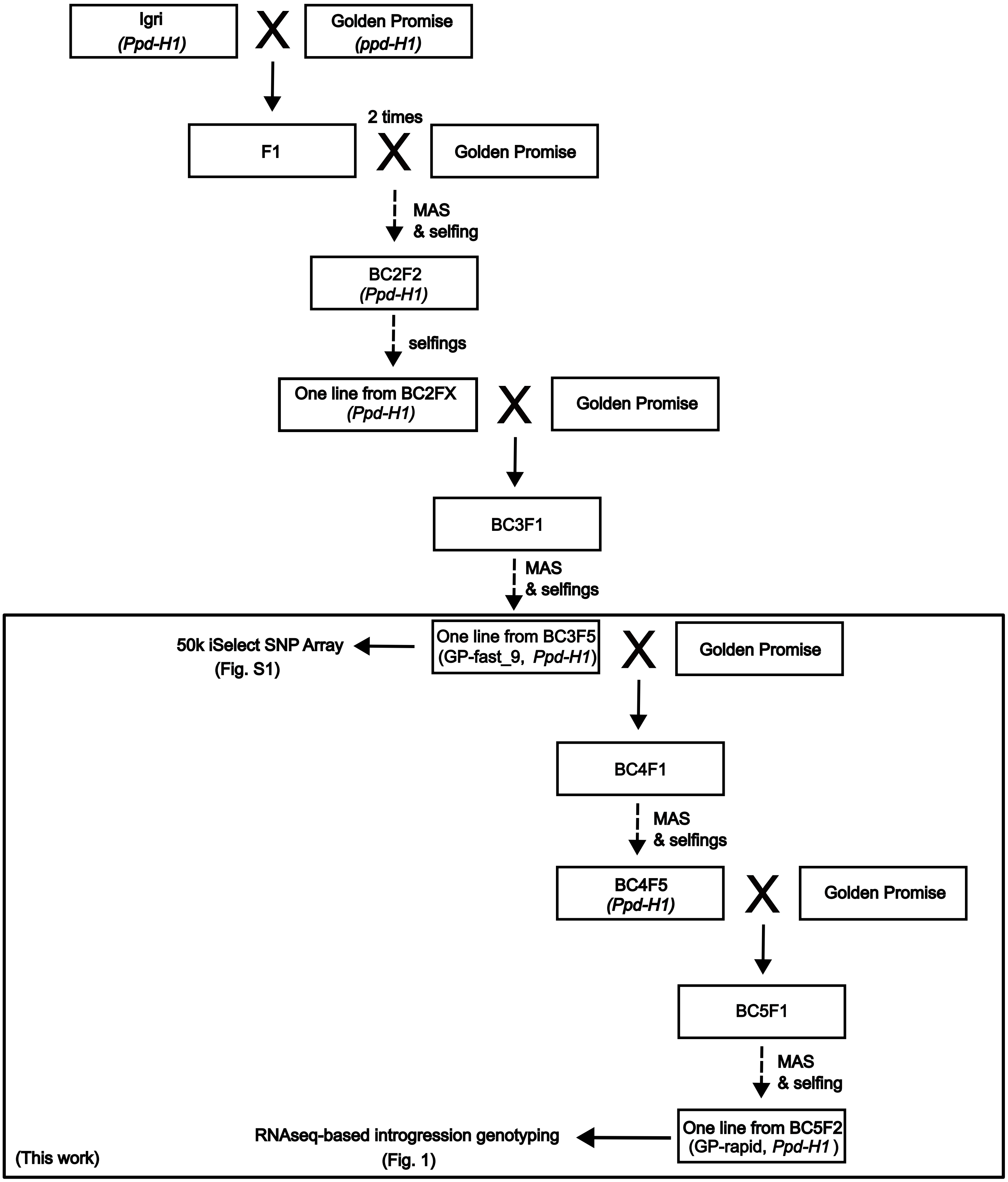

Fig. S2. Graphic overview of GP-rapid generation.
